# Energy Transition in Northern Netherlands: seeking a balance between top-down and bottom-up initiatives

**DOI:** 10.12688/openreseurope.17431.2

**Published:** 2024-10-28

**Authors:** Bas Oldenbroek, Ifigenia Psarra, Tineke van der Schoor, Cyril Tjahja

**Affiliations:** 1 Research Center for the Built Environment NoorderRuimte, Hanze University Groningen, Groningen, Groningen, 9747 AS, The Netherlands; 2 Faculty of Humanities, Art and Culture, History, Antiquity, Vrije Universiteit Amsterdam, Amsterdam, North Holland, The Netherlands

**Keywords:** energy transition, citizen engagement, local energy initiatives, bottom-up processes, balanced approach, Groningen, thematic analysis

## Abstract

This brief report focuses on top-down and bottom-up processes within the field of energy transition. It aims at gaining a better understanding of the needs of the local energy initiatives. On this basis, policy recommendations are formulated to help the municipality of Groningen to facilitate local energy initiatives, ultimately leading to a more balanced approach of the local energy transition. Thus, this study explored the (mutual) interests, barriers and expectations of the municipality and local citizen initiatives. The theoretical framework is the implementation analysis framework, distinguishing top-down and bottom-up approaches. Specifically, this qualitative (thematic analysis) research study investigates the mismatch in expectations between a number of local energy initiatives and the municipality of Groningen regarding their roles within the local energy transition context. To this end, semi-structured interviews have been conducted with members of the municipality of Groningen, Grunneger Power (a local energy intermediary), and four local energy initiatives. Need and expectation gaps have been identified and potential solutions have been explored. The main findings of the study illustrate the need of professional support for citizen initiatives, at both technical and organizational level, especially in the first phases of their development. Additionally, clear mutual communication on short and long-term planning and ambitions of the involved parties is of key importance for the alignment of the interests and the course of actions. Consequently, a clear context is needed, within which an exchange of feedback on the envisioned strategies, and the subsequent energy saving or generation interventions, can take place in an efficient and effective way. Additionally, such a context increases confidence and provides a clear understanding to the citizen initiatives regarding their role and the level and nature of support they can expect in their intended projects and activities. Based on these findings, policy implications have been drawn.

## Introduction

The theoretical framework of this study is the implementation analysis framework ( Sabatier & Mazmanian, 2005), according to which, two transition processes are distinguished: the ones emerging from a top-down, and those stemming from a bottom-up approach. To catalyse the energy transition, there is a need to broaden the commonly adopted strictly top-down approach, by embracing and fostering the roles that local government, entrepreneurs and citizens can play together ( Allen *et al.*, 2012) ( Hargreaves *et al.*, 2013) ( Klösters *et al.*, 2020) ( Psarra *et al*., 2024). Furthermore, several authors discuss the role of intermediaries or ‘middle actors’, for example ( Parag *et al*., 2013; Parag & Janda, 2014) find that intermediary organizations play a crucial role in the transfer of information to local energy organizations. They also point to the importance of capacity building for local energy activities.

In the Netherlands, numerous local grassroot energy initiatives have emerged with the goal of accelerating the local energy transition ( Rotmans, 2011). There has been an enormous increase in the number of Local Energy Initiatives (LEI)s over the last seven years ( HIER; RVO, 2021). In 2020, there were a total of 623 LEIs in almost every municipality in the Netherlands. Specifically, LEIs are defined as: "A group of citizens who collectively, with the intention of collaborating on making the local energy supply more sustainable and taking initiatives to that end. This includes activities aimed at energy generation, energy conservation, energy supply, collective purchasing or commissioning and other energy-related activities. The collective focuses on its own living environment and community (neighbourhood, village, city or region) and combines energy goals with social, societal and economic goals." ( HIER; RVO, 2021)

In previous research conducted by the Dutch Organisation for Applied Scientific Research (TNO), insight was gained into the role of local initiatives in the energy transition, and the steps they go through when carrying out their activities ( Klösters *et al.*, 2020). They focused on ten LEIs spread over different municipalities in the Netherlands. Various success factors and barriers that arose during this process have been formulated. A large number of these barriers relate to the role of the municipality in facilitating the activities of the local initiatives. Examples vary from barriers in legislation and regulations to diverging interests between local initiatives and the municipality.

Despite the growth in the number of LEIs, the impact of these collectives on the national and international climate objectives is still limited ( Wiekens & Germes, 2020). LEIs and top-down energy planning procedures rarely align. The practical studies that are mentioned within the literature review on LEIs, show that the role of the municipality in facilitating LEIs is often dominant and top-down oriented ( Klösters *et al.,* 2020; van der Windt *et al.,* 2021). However, according to Sabatier & Mazmanian (2005), a more balanced approach, taking into account the needs, interests, but also the barriers in the relationship between local energy initiatives (LEIs) and municipalities could accelerate citizen engagement and result in more successful local energy transition projects.

This brief report offers policy recommendations to help the municipality of Groningen to facilitate and better understand the needs of local energy initiatives, ultimately leading to a more balanced approach of the local energy transition. Thus, this study explored the (mutual) interests, barriers and expectations of the municipality and local citizen initiatives. The case study area is the Groningen municipality, which is located in the North of the Netherlands. Groningen is one of the two lighthouse cities participating at the Making City Horizon 2020 research program, in the context of which this study has been conducted. The various districts of Groningen are characterized by different stages of energy transition, as well as by a variety on the ways with which citizen engagement is approached. In some districts, LEIs have been formulated by residents, while in other districts they have been established and facilitated by the municipality. It is interesting to notice that usually the vision and the interests of these LEIs do not exclusively revolve around the theme of energy transition, but are broader. For instance, they usually relate to enhancing the overall well-being and the quality of life of the local residents, or making the district more sustainable. Then, energy transition usually constitutes an important aspect within this broader frame of interests and ambitions. Finally, the variety among the districts in terms of sociodemographics and characteristics of the built environment implies a different set of challenges and needs within the context of energy transition, and thus the need for different approaches on encouraging citizen engagement and supporting a local LEI.

In the following, the methodology that has been followed in this study is described, the results are then discussed and conclusions in the form of recommendations are drawn.

## Methods

Qualitative data were collected through semi-structured interviews conducted between October 2021 and January 2022. These interviews involved a member of the municipality of Groningen, a representative of the local energy intermediary Grunneger Power, and representatives from four Local Energy Initiatives (LEIs) ( Table 1). A graduating student from the multidisciplinary Master of Science program ‘Energy for Society’ at Hanze University of Applied Sciences in Groningen, the Netherlands, conducted the interviews under the supervision of the co-authors.

**Table 1.** Overview of the LEIs that participated in this study.

*Inteviewed LEI*	*Role of interviewee*	*District in Groningen*	*Level of LEI’s experience*	*Key activities*
***LEI A***	Founding member	Zeehelden buurt	Unexperienced (±2 years)	Making the district more sustainable and greener.
***LEI B***	Co-founder	Paddepoel	Experienced (±10 years)	Making houses in the district more sustainable.
***LEI C***	Founding member	Hoogkerk	Unexperienced (±1 year)	Making the neighborhood more sustainable within the themes of green, food, energy and culture.
***LEI D***	Chairman	Noorder plantsoen buurt	Experienced (±8 years)	Making the neighborhood aware of the energy transition.
***Intermediary X***	Initiative and process facilitator	All districts of Groningen	Experienced (±10 years)	Promoting energy transition and sustainability by helping LEIs and other corporations, and by setting up participation projects in the municipality of Groningen.

It was intended to interview representatives of Local Energy Initiatives (LEIs) with diverse profiles, including various levels of maturity, years of existence, scopes of interest, and types of key activities. All the LEIs are active in the city of Groningen. As this is a qualitative study, the aim was not to achieve a large sample size, but rather to engage with key representatives from the most active and relevant LEIs in the city. Specifically, the case study focused on the municipality of Groningen, and given the limited number of LEIs in the area—approximately six in total—the four interviews conducted offer a representative view of their needs and expectations. In addition, speaking with a representative from the municipality and with a representative of Grunneger Power, which is a large and experienced local energy initiative that also acts as an intermediary for the other, smaller LEIs in the municipality, this sample was considered sufficient to cover the central topics related to local energy initiatives.

The interviewed representatives were highly engaged and provided valuable insights into how their respective LEIs interact with local society and the municipality. During the interviews, the needs and expectations gap between the LEIs and the municipality of Groningen regarding the LEIs role within the local energy transition context were discussed. Furthermore, potential solutions for bridging that gap have been explored.

Each interview lasted approximately one hour and was conducted in Dutch, except for the interview with the municipality representative, which was conducted in English. Due to COVID-19 restrictions, the interviews were conducted online via Microsoft Teams. The sessions were recorded and transcribed using the transcription tool Sonix.ai ^1^. The collected data were then anonymized, and any transcription errors were corrected. The transcripts are available on the DataverseNL data repository ( Psarra, 2024).

### Data analysis

The collected data have been analysed with the thematic analysis method, by using the ATLAS.ti (version 7) qualitative data analysis tool ^2^. First, open coding was performed (data being marked and labelled with a summary name), resulting in a list of codes. Next, code clusters emerged, by implementing axial coding. Then, the underlying connections and patterns were established in the form of five themes in line with the step-by-step method identified by Braun and Clarke (2006).

## Results

Our findings highlight the challenges and needs faced by LEIs as they navigate their roles and responsibilities. The thematic analysis has revealed five key areas of focus: the need for professional support and the varying degrees of professionalisation, which are mostly related to the internal organisation of LEIs; the impact of mutual ignorance leading to mismatched expectations, as well as the necessity for clarity in roles and responsibilities, which mainly refer to the lack of sufficient communication between the LEIs and municipality; and the importance of effective communication, which refers to ways of improving the latter collaboration.

Below, each of these themes is discussed in detail. 

### Need for professional support

Since LEIs consist of volunteers of varying degree of knowledge and experience, there is a great demand for professional support on both organizational and practical levels. A member of the city council mentioned in the interview:

*“But citizens don't have the power or the time or expertise to do collective projects. And they actually need some support from the municipality or from another party like, well, could also be academia or a party like Grunneger Power to help to develop those ideas. The citizens are waiting for, well, either more guidance from the municipality or from the country also. I mean, it's not the municipality, it's also the national government who is lagging behind with legislation. But basically, they [the citizens] are waiting for somebody higher up and higher up is waiting for citizens to take their own initiative, basically.”*

Especially in their initial phase, most LEIs seem to struggle with organizational and practical matters. One of the LEIs states:

*“To stimulate initiatives, it would be very nice if there is already some kind of plan or some kind of signpost, a route plan like: ‘If you are going to set up an initiative, you have to take this, this and this into account, a kind of manual for local initiatives”.*

Some LEIs indicate that there is a need for a facilitating party, such as Grunneger Power, Groninger Milieufederatie and GrEK, with experience in supporting other initiatives that have encountered similar problems in the past. For instance, GrEK already gains a lot of experience within the role of the intermediary, offering lots of support to its members. Specifically, GrEK is more orientated towards the supply of support to initiatives in the province of Groningen, while Grunneger Power mostly focuses on the LEIs of the city of Groningen. Thus, it is also reasonable that the LEI member who has been interviewed has not been aware of the support and the courses that GrEK currently offers. In addition to organizational knowledge, support regarding practical activities is also deemed to be necessary. Furthermore, the need for technical support (such as contractors and installers) is expressed, as well as the need for a fixed municipal representative with whom a LEI can build a long-term relationship of trust. Finally, in addition to the aspects of professional support mentioned above, funding remains one of the basic needs of LEIs.

### The desired degree of professionalization

The desired degree of professionalization varies among the LEIs. Several LEIs express the ambition of becoming more professional, by indicating aspects such as 'public relationships', 'a newsletter' or 'the opportunity to train someone internally'. However, there are also several LEIs emphasizing their volunteer nature, whilst some of those observe an implicit requirement from the municipality for them to professionalise.

*"We just do it as volunteers, little by little in the evenings, a little bit at the end of the working day...In our group, everyone works full-time, or almost full-time, so yes, we have sometimes better things to do, so that also makes it very vulnerable."*

On the other hand, a representative of the municipality explained:

*“Actually, because we have all sorts of initiatives and corporations dealing with all sorts of topics, and some are more happy by not having the municipality involved that much. Some really don't know what to do at some point, so there is not really something to discuss about. But at a certain moment when there is something that they want to discuss or need to discuss, then it is something that they should reach out and say, OK, now we are at this moment and now we should really talk about how to make this into a more solid plan.”*

### Mutual ignorance creates mismatched expectations

The LEIs indicate that they need a suitable project leader from the municipality who is aware of their vision and with whom they can collaborate. Both parties have expectations, which are to a great extent based on assumptions. Indicatively, municipalities set certain goals, for example being CO2 neutral by 2035, and they want to achieve these goals within a particular timeframe. On the other hand, LEIs often aim to trigger citizen engagement on a district level and are less focused on particular timeframes:

*“The municipality is very much geared to programs and initiatives are not. Initiatives sometimes float there and then there again. And those two cultures don't work well with each other.”*

The mutual ignorance between the municipality and LEIs causes mismatched expectations, which appear to slow down the local energy transition process. On the one hand, LEIs expect the municipality to take the lead by giving them a clear role within the energy transition process, and by actively facilitating them. On the other hand, the municipality seems to have a wait-and-see attitude towards LEIs and to expect them to become more professional before concrete responsibilities are delegated to them regarding local energy transition. Indicatively, a representative of the municipality explained in his interview:

*“If they have specific questions, then we will help them with that. And perhaps because when it comes to the infrastructure, they cannot do that by themselves. Yeah, and then it depends on when good time to do so and when they want to develop solar initiatives, they can better work together with Grunneger Power and the municipality or with some other corporation.”*

### Need for clarity

It is important for LEIs to have a clear picture about which tasks and responsibilities are allocated to them. For instance, LEIs indicate that there is a need for (timely) clarity about whether their project ideas are considered feasible and realistic by the municipality. It is also indicated that there is a need for a more detailed municipal plan for the various districts. This would create more clarity about how the LEIs can contribute to the municipal goals for the corresponding districts. LEIs aim at being regarded as a serious partner within the local energy transition context, for instance by participating in the formulation of the municipal district energy plans. These results tie in with the recommendations of TNO (2020), regarding the need for common goals and a clear position for LEIs. A LEI representative mentioned in her interview:

*“I mean, does this mean that we as citizens should start our own plans? And for example, if we want to make a local heat storage, that we as citizens have to do this? Or does it mean that there is still a possibility that we can get support from the municipality for doing this?”*

### The importance of communication and collaboration

A lack of sufficient communication between LEIs and the municipality received also special attention during the interview sessions. For instance, the need for a platform on which information on district level about e.g. energy consumption data would be quite helpful for LEIs.

Additionally, there is also a need for communication and collaboration among the various LEIs, as they might often be engaged in similar activities or face similar problems (e.g. during the initial phase). In the C *ustomer Journey of Collectives* research, TNO (2020) also refers to insufficient knowledge sharing between LEIs. One of the interviewed LEIs indicates:

*“And on the other hand, I think, if there is a neighbourhood initiative, to facilitate that in such a way that neighbourhood initiatives come into contact with each other to learn from each other, to avoid having to reinvent the wheel.”*

## Conclusions

To bridge the gap between the municipality and the LEIs, in the context of professional support, the following recommendations were formulated. First, the development of a professional support framework for LEIs by the municipality, in which the role of intermediary organizations is specified. In particular, regional or city scale energy co-operatives or organizations (such as “Energie van Ons” and “GREK”), which can play an intermediary role between the municipality and grassroot initiatives, can be beneficial for achieving a balance between top-down and bottom-up processes in local energy transition. That is also found in van der Schoor & van der Windt (2023). Various LEIs profiles can be considered, to allow for flexibility regarding the content and the ways that this support is provided. In addition, the creation of a clear municipal point of contact for LEIs is also important.

Secondly, it is crucial for both the municipality and Local Energy Initiatives (LEIs) to have a mutual understanding of their roles within the local energy transition context. LEIs should effectively communicate their vision and mission to the municipality. Intermediaries can facilitate the shaping of these ambitions and the subsequent needs for municipal support. Moreover, mutual agreements between each LEI and the municipality about the degree of professionalization expected by them, in relation to their vision and mission, can be very beneficial.

Thirdly, a collaboratively developed roadmap outlining the citizen engagement approach and the role of the corresponding LEI can be integrated into the district energy plans formulated by the municipality ( Gemeente Groningen, 2020). Specifically, local district energy plans, characterized by a place-based approach, can serve as the foundation for a tailored roadmap. This roadmap can be customized according to the capacity and expertise available within the corresponding citizen initiatives and the specific socio-spatial context of the districts. Moreover, in developing these plans, LEIs can play a catalytic role by gathering and incorporating input and viewpoints from a broader segment of the local community, beyond just LEI members ( Psarra *et al*., 2024). Finally, the collaboration and knowledge sharing between LEIs, e.g. via structural consultations among various LEIs representatives, the municipality and intermediary organisations, should be encouraged.

## Ethics and consent

The Hanze Ethical Advice Committee (HEAC) of the Hanze University of Applied Sciences has provided their approval to the retrospective review request for this research study. The committee used the Assessment Criteria Ethical Advice Committee of Hanze University of Applied Sciences. They approved the review request on the 7th of February of 2024. The approval number is: heac.T2023.050. The reason for requesting the review retrospectively was that it is a non-medical, low-risk research study.

Before starting each interview, we obtained oral consent from participants to record the interview and publish the findings. To obtain Ethical approval, we needed to submit written consents as well. Therefore, we contacted the participants afterward to sign the written consent forms.

## Data Availability

DataverseNL: Interviews with Local Energy Initiatives (LEIs) representatives. https://doi.org/10.34894/118BLP ( Psarra, 2024). This project contains the following underlying data: Interview transcripts This project contains the following extended data: Semi-structured interview scheme Code clusters, as they emerged during the data analysis process Data are available under the terms of the Creative Commons Attribution 4.0 International license (CC-BY 4.0).
