# Supplementary material for: Energy Transition in Northern Netherlands: seeking a balance between top-down and bottom-up initiatives
Source: Open Res Eur. 2024 Oct 28;4:139. Originally published 2024 Jul 9. [Version 2] doi: 10.12688/openreseurope.17431.2 (PMC11739859; doi:10.12688/openreseurope.17431.2)
Supplement: Peer review history

# Reviewer report: Reviewer response for version 2

**DOI:** 10.21956/openreseurope.20270.r48327

**Published:** 2025-01-17

**Authors:** Benjamin Schmid, Ange Nkonko Kibelo Martin's

**Affiliations:** 1 Institute of Geography and Sustainability, University of Lausanne, Lausanne, Switzerland

We would like to express our gratitude to the authors for their efforts in conducting this study. Better understanding and improving relationships between emerging local energy initiatives and municipalities is an important area of study to facilitate democratic energy transitions, not only in the Netherlands, but across Europe and globally. The study examines the expectations of various local initiatives, as well as the municipality and an intermediary in the city of Groningen, regarding their cooperation in the local energy transition. Although the study offers interesting insights into these dynamics, we see the need for a comprehensive review before we can recommend final indexing of the manuscript. 

 Our core issue, echoing in further questions, concerns the main purpose of the article. We are aware that the “short report” format differs from articles in other journals, as there is less space available, and the report character is more pronounced. However, the manuscript is currently written as if it is somewhere between a research article and a policy brief, which makes it ambivalent. Is the purpose of the analysis to identify and formulate specific recommendations for the situation in Groningen, or is the main purpose to provide a basis for more general findings (in the sense of a research article)? While the introduction emphasizes the former, the conclusion begins with a more general statement, indicating an ambition towards the latter purpose. The key purpose should be made clearer and less ambivalent. 

 If the manuscript is to be read as a research article, we have found that the embedding of the research interest in the established body of literature is underdeveloped, particularly in terms of the lack of a clear research gap relevant to the academic literature.  

 Furthermore, the application of Sabatier & Mazmanian’s *implementation analysis framework *as theoretical framework in the analysis seems both opaque and not entirely accurate: 

- The framework is only briefly mentioned in the introduction, while it remains unclear whether and how it was applied as an analytical framework in the analysis. The fact that “open coding was performed…» (p. 4) as the first step in the data analysis suggests that the framework was not used in any major analytical capacity. It is also not mentioned in the discussion or conclusion to make sense of the findings. 

- It seems questionable to us how appropriate the framework is for the present study (assuming that the authors refer to the study/framework published in 1985 - and not 2005 as written). First, the framework was intended to be applied to policy implementation processes and not necessarily to transitions. Second, the terms “top-down” and “bottom-up” (notably in the title of the manuscript) are not entirely clear and seem to be interpreted differently than suggested in Sabatier & Mazmanian's framework. In the original framework, as we understand it, the difference between top-down and bottom-up refers to the *analytical perspective* (with bottom-up referring specifically to a focus on the local implementation process, including what happens at the local government and administrative level; whereas municipal action is understood as top-down in the manuscript). In contrast, the authors seem to understand the terms “top-down” and “bottom-up” to refer to different *governance approaches* to implementation itself and assume a current imbalance in favour of top-down approaches that should be more balanced in order to accelerate the energy transition. While this is a common assertion in the energy transition literature, it is hardly a direct claim of the policy implementation framework (there is a misleading indirect quote on p. 3 that Sabatier & Mazmanian have made such a claim).  

In light of these issues with the framework, we suggest either dropping it altogether or making it clearer how it has been applied analytically and describing more explicitly the national/top-down policies whose implementation is being examined. In any case, the authors should make it clearer what they mean by bottom-up and top-down (processes). 

 Furthermore, if understood as a research article aiming to make more general statements, it would be desirable in the area of analysis/methodology to learn more about how the situation in Groningen is representative of situation elsewhere - why it is an appropriate case? Also, we wondered, firstly, why only the most active and relevant LEIs were interviewed (wouldn't the non-active LEIs also have been of interest to investigate the research question), and secondly, how the identified differences between the LEIs were reflected analytically (levels of maturity, municipality as initiator or not). How did these differences influence the statements and results on the individual LEIs? At present, the studied LEIs are treated rather homogeneously. Finally, we asked ourselves how representative the interviewees are of the organizations they speak for. While a single interviewee may be sufficient for the LEIs, it remains questionable whether the statements of a single person from the municipality (who apparently does not speak Dutch well, which notably may have complicated the exchange with the LEIs) sufficiently reflect the views and interests of the ‘city’. A critical discussion on these points would greatly improve the analysis (and also the robustness of the policy recommendations), maybe instead of mentioning all the software used.  

 With regard to the results, we found the direct quotes selected from the interviews to be well chosen and meaningful. We would also like to positively acknowledge that the interview transcripts were published. But we would have hoped for a little more (critical) discussion when interpreting the results/statements from the interviews. This especially concerns not attributing different views between LEIs and the municipality solely to a lack of knowledge/mutual ignorance but considering the fact that these actor types operate with different institutional logics, responsibilities, and with accountabilities to different publics. Linked to this point, we would find a revision of Table 1 desirable: instead of “key activities”, it would be more helpful for the analysis to present the ‘key objectives, as these appear to us to be more relevant for differences in expectations. Consequently, it would also be helpful to add the municipality as another actor and to mention its relevant key objectives / policies.  

 Many of our points are particularly important if the article is to be read as a research article. But we also see them as helpful if the focus is primarily on formulating policy recommendations.

If applicable, is the statistical analysis and its interpretation appropriate?

Not applicable

Are all the source data and materials underlying the results available?

Yes

Is the study design appropriate and is the work technically sound?

Yes

Is the work clearly and accurately presented and does it engage with the current literature?

Partly

Are the conclusions drawn adequately supported by the results?

Partly

Are sufficient details of methods and analysis provided to allow replication by others?

Not applicable

Reviewer Expertise:

Community energy, energy democracy, citizen participation

We confirm that we have read this submission and believe that we have an appropriate level of expertise to confirm that it is of an acceptable scientific standard, however we have significant reservations, as outlined above.

# Reviewer report: Reviewer response for version 2

**DOI:** 10.21956/openreseurope.20270.r48335

**Published:** 2025-01-15

**Authors:** Adolf Acquaye

**Affiliations:** 1 Khalifa University, Abu Dhabi, Abu Dhabi, United Arab Emirates

- The report is clearly presented and engages with current literature, citing relevant studies and frameworks.
- The study design, which includes semi-structured interviews and thematic analysis, is appropriate and technically sound for the research objectives.
- While the methodology section provides a good overview, more detailed descriptions of the interview process and data analysis steps could enhance replicability. Hence details of methods and analysis for replicability is evaluated as **PARTLY**.
- The report mentions that transcripts are available on the DataverseNL data repository, which supports transparency and data availability.
- The conclusions are well-supported by the findings from the thematic analysis and the interviews conducted.
- The report is based on qualitative research, so statistical analysis is not applicable

If applicable, is the statistical analysis and its interpretation appropriate?

Not applicable

Are all the source data and materials underlying the results available?

Yes

Is the study design appropriate and is the work technically sound?

Yes

Is the work clearly and accurately presented and does it engage with the current literature?

Yes

Are the conclusions drawn adequately supported by the results?

Yes

Are sufficient details of methods and analysis provided to allow replication by others?

Partly

Reviewer Expertise:

Sustainability Research

I confirm that I have read this submission and believe that I have an appropriate level of expertise to confirm that it is of an acceptable scientific standard.

# Reviewer report: Reviewer response for version 2

**DOI:** 10.21956/openreseurope.20270.r48334

**Published:** 2025-01-06

**Authors:** Paola D’Orazio

**Affiliations:** 1 Technische Universität Chemnitz,, Chemnitz, Germany

The manuscript presents a comprehensive study on the role of Local Energy Initiatives (LEIs) in the energy transition of Groningen, offering a well-structured and detailed investigation.

 The introduction effectively sets the stage by putting the research within the theoretical framework of the Sabatier and Mazmanian implementation analysis framework, distinguishing between top-down and bottom-up approaches. The discussion emphasizes the need to foster collaboration among local governments, entrepreneurs, and citizens to catalyzes energy transitions. While the introduction is thorough and well-referenced, it could benefit from further clarification on how this study builds upon or diverges from previous research in the field. For example, the definition of LEIs is insightful, yet comparing these with similar citizen-driven initiatives elsewhere could enhance understanding. 

 Some references, such as those to HIER and RVO (2021), are mentioned but not fully integrated into the narrative, leaving gaps in their contribution to the overall argument.

 The methodology section is clearly described, outlining the use of semi-structured interviews with representatives from LEIs, the municipality, and an intermediary organization. 

 The small sample size is justified as sufficient for qualitative analysis, but further explanation of its representativeness and the rationale behind selecting these specific LEIs would strengthen the section. 

 The use of thematic analysis with the ATLAS.ti tool is appropriate, but the manuscript would benefit from a brief explanation of why this method and software were chosen over alternatives.

 The findings are well-organized around key themes, including professional support needs, mutual ignorance between LEIs and municipalities, and the importance of communication and collaboration. These themes are supported by direct quotations, which effectively highlight the perspectives of participants. However, certain findings, such as the impact of "mutual ignorance," could be better illustrated with concrete examples to clarify their implications. Similarly, the discussion on professionalization among LEIs is insightful but would benefit from further exploration of how differing levels of professionalization impact their ability to collaborate with municipalities effectively.

 The manuscript provides thoughtful policy recommendations to bridge the gap between municipalities and LEIs. These include the development of a professional support framework, a clearer mutual understanding of roles, and tailored roadmaps for citizen engagement. While these recommendations are valuable, they could be more detailed and actionable. For instance, the proposal for a professional support framework is promising, but specific examples of successful implementation from other municipalities or countries would add depth and practical relevance.

 The conclusion highlights the importance of collaboration and knowledge sharing, but it would benefit from addressing potential challenges, such as resource limitations or stakeholder resistance, that might arise in implementing the proposed strategies.

If applicable, is the statistical analysis and its interpretation appropriate?

Not applicable

Are all the source data and materials underlying the results available?

Yes

Is the study design appropriate and is the work technically sound?

Yes

Is the work clearly and accurately presented and does it engage with the current literature?

Partly

Are the conclusions drawn adequately supported by the results?

Yes

Are sufficient details of methods and analysis provided to allow replication by others?

Partly

Reviewer Expertise:

climate risks, sustainable energy transitions

I confirm that I have read this submission and believe that I have an appropriate level of expertise to confirm that it is of an acceptable scientific standard, however I have significant reservations, as outlined above.

# Reviewer report: Reviewer response for version 2

**DOI:** 10.21956/openreseurope.20270.r48330

**Published:** 2025-01-03

**Authors:** Md Tasbirul Islam

**Affiliations:** 1 Interdisciplinary Research Center for Sustainable Energy Systems (IRC-SES), King Fahd University of Petroleum and Minerals (KFUPM), Dhahran, Eastern Province, Saudi Arabia

I read the article and found very useful for broader energy transition-related academic discourse. 

 I am satisfied with the approach that the authors taken towards the research method and illustrations of the findings in a clear and concise manner. Within the scope of a brief report, it delivered key messages from the ground level and I encourage the authors in-depth analysis in future towards a full-length journal article. 

 Thank you.

If applicable, is the statistical analysis and its interpretation appropriate?

Not applicable

Are all the source data and materials underlying the results available?

Yes

Is the study design appropriate and is the work technically sound?

Yes

Is the work clearly and accurately presented and does it engage with the current literature?

Yes

Are the conclusions drawn adequately supported by the results?

Yes

Are sufficient details of methods and analysis provided to allow replication by others?

Yes

Reviewer Expertise:

Sustainability, Circular Economy, Life Cycle Assessment, Energy Policy, Waste Management, Material Flow Analysis

I confirm that I have read this submission and believe that I have an appropriate level of expertise to confirm that it is of an acceptable scientific standard.

# Reviewer report: Reviewer response for version 2

**DOI:** 10.21956/openreseurope.20270.r47824

**Published:** 2024-12-14

**Authors:** Lefteris Topaloglou

**Affiliations:** 1 University of Western Macedonia (UOWM),, Kozani, Greece

The paper, *"Energy Transition in Northern Netherlands: Seeking a Balance Between Top-Down and Bottom-Up Initiatives,"* examines the interplay between local energy initiatives (LEIs) and municipal authorities within the energy transition process in Groningen, Netherlands. The study employs the implementation analysis framework to understand gaps in expectations and needs between the two parties. The article offers policy recommendations to address these challenges, emphasizing the importance of mutual understanding, professional support, and knowledge sharing.

- The framework Sabatier and Mazmanian is introduced without a clear rationale, and its application is not deeply integrated into the analysis. Please provide detailed justification for using this framework. Explain its relevance compared to alternatives and clarify how the framework informed data collection and analysis.
- The methodology section lacks detail about the thematic analysis. Please expand the methodology to explain the coding process, the tools used, and steps for ensuring reliability. Furthermore, discuss the strengths and limitations of using a small sample size for qualitative research.
- Please relate findings explicitly to the theoretical framework and existing studies on energy transitions and governance, aiming to strengthen the linkage with the theoretical discussion.
- The recommendations are practical. However, it would be interesting to elaborate on how these recommendations could be adapted to different socio-spatial contexts beyond Groningen.
- Suggest future research directions, such as comparative studies in other municipalities or quantitative analyses of LEI impacts.

Addressing these points will significantly improve the article's scientific validity and impact.

If applicable, is the statistical analysis and its interpretation appropriate?

Yes

Are all the source data and materials underlying the results available?

Partly

Is the study design appropriate and is the work technically sound?

Yes

Is the work clearly and accurately presented and does it engage with the current literature?

Partly

Are the conclusions drawn adequately supported by the results?

Yes

Are sufficient details of methods and analysis provided to allow replication by others?

Yes

Reviewer Expertise:

just energy transition, spatial justice, place-based approaches and analysis, governance of energy transition

I confirm that I have read this submission and believe that I have an appropriate level of expertise to confirm that it is of an acceptable scientific standard, however I have significant reservations, as outlined above.

## References

[1] : Energy Transition in Northern Netherlands: seeking a balance between top-down and bottom-up initiatives. *Open Research Europe* .2024;4: 10.12688/openreseurope.17431.2 10.12688/openreseurope.17431.2 39830892 PMC11739859

# Reviewer report: Reviewer response for version 2

**DOI:** 10.21956/openreseurope.20270.r46608

**Published:** 2024-11-22

**Authors:** Ruth Shortall

**Affiliations:** 1 Joint Research Centre (JRC), Petten, The Netherlands

This report describes a qualitative study aimed at deriving policy recommendations regarding the needs of local energy initiatives.  It aims to provide insights into the mutual interests, barriers and expectations of the municipality and local citizen initiatives.  Given the benefits of qualitative approaches in analysing policy outcomes in socio-technical systems, the results are likely of value for policy-makers at various levels and so are worthwhile to publish.  As it stands, the text would not be sufficient if submitted as a journal article, however, I appreciate that the brief report format limits the authors. Nonetheless, certain (concise) additions would improve the report:

- It appears the analytical framework for the qualitative data is mainly based on the work on Sabatier & Mazmanian (2005). This appears to have been added as somewhat of an afterthought. Given the lack of theoretical background, it is not clear why the theoretical framework of Sabatier and Mazmanian was chosen, and not others?  What makes this framework special, or more suitable for this study?
- What kind of thematic analysis was carried out exactly? It would be good to specify if deductive or inductive coding was used
- It is not mentioned whether or not, or how, the codes were checked for consistency
- The pros and cons of using a qualitative approach could be elaborated – what do we miss and gain from having a small but very rich data set?
- A bit more discussion and reflection, comparing and contrasting results to other studies, aside from those of TNO, would situate the results of this study more clearly in the existing broader literature. What do we learn overall and how do the results tie into the bigger picture of energy transition governance? Or energy justice? 
- What are the limitations of this study and how might these impact on the use of the results by policy-makers?

If applicable, is the statistical analysis and its interpretation appropriate?

Not applicable

Are all the source data and materials underlying the results available?

Yes

Is the study design appropriate and is the work technically sound?

Yes

Is the work clearly and accurately presented and does it engage with the current literature?

Partly

Are the conclusions drawn adequately supported by the results?

Yes

Are sufficient details of methods and analysis provided to allow replication by others?

Partly

Reviewer Expertise:

energy justice, socio-technical policy appraisal, citizen engagement

I confirm that I have read this submission and believe that I have an appropriate level of expertise to confirm that it is of an acceptable scientific standard, however I have significant reservations, as outlined above.

# Reviewer report: Reviewer response for version 2

**DOI:** 10.21956/openreseurope.20270.r45941

**Published:** 2024-11-21

**Authors:** Mahyar Kamali Saraji

**Affiliations:** 1 Kaunas Faculty, Vilnius University, Kaunas, Lithuania

This research emphasizes both top-down and bottom-up processes related to energy transition. Its goal is to gain a deeper insight into the requirements of local energy initiatives. The top-down versus bottom-up strategies in energy transition are authoritative in the present endeavors toward sustainable development. The authors offer tangible solutions to policymakers by identifying the needs and barriers to LEIs that can foster better energy governance. The overall presentation of the study is also acceptable. 

 It extends useful information and suggests policies based on its findings, but its academic discipline can be significantly enhanced. There are some limitations regarding the work's relationship with the article's body of literature. The model developed by Sabatier and Mazmanian has been mentioned, but implementation analysis is not featured in the case study design or findings. It would be desirable to elaborate on such a framework, linking it to the more general discussions on the roles of intermediaries and LEIs that complement the paper.

 The study's sample size of six interviews is relatively small in terms of qualitative research for which the study design is appropriate. Also, there is a need for more clarity about the data analysis, for example, how the codes came into existence and how they were tested, which were neglected in the study.

 Although the recommendations for bridging the gaps between the LEIs and the municipality are very convenient, their relation with the findings needs more clarity and explanation. For instance, the argument that LEIs should design a roadmap could have been better substantiated with specific evidence or examples from the analysis conducted. It would also be beneficial to the overall impact of the paper to discuss the implications of these findings in terms of energy policy in other countries and other contexts. 

 To summarize, the study addresses a significant gap and has implications for policymakers, yet it does not make an academic contribution to the field. Greater integration of theory with empirical work, better methodological clarity, and a more coherent presentation of the findings would improve the quality of the work.

If applicable, is the statistical analysis and its interpretation appropriate?

Not applicable

Are all the source data and materials underlying the results available?

Partly

Is the study design appropriate and is the work technically sound?

Partly

Is the work clearly and accurately presented and does it engage with the current literature?

Partly

Are the conclusions drawn adequately supported by the results?

Partly

Are sufficient details of methods and analysis provided to allow replication by others?

Not applicable

Reviewer Expertise:

Low-carbon energy transition, energy economics, and sustainability

I confirm that I have read this submission and believe that I have an appropriate level of expertise to confirm that it is of an acceptable scientific standard, however I have significant reservations, as outlined above.

# Reviewer report: Reviewer response for version 1

**DOI:** 10.21956/openreseurope.18838.r42526

**Published:** 2024-08-23

**Authors:** Lorenzo De Vidovich

**Affiliations:** 1 Università di Trieste, Trieste,, Italy

The paper focuses on the interplay between top-down and bottom-up processes within local energy transition initiatives. It aims at gaining insights into the ways that a more balanced approach can be achieved, by taking into consideration the (mutual) interests, barriers and expectations of the municipality and local citizen initiatives. Needs and expectation gaps in local projects have been identified and potential solutions have been explored relying on qualitative outcomes and on the "implementation analysis framework" as a theoretical framework, merely introduced to distinguishing top-down and bottom-up approaches, without a deepened and well-informed theoretical section. As a first comment, I therefore suggest to reframe a bit the theoretical framework to make it more effective, even using more references from Paul Sabatier or to other words that investigated the combination between top-down and bottom-up approaches.

**Further comments**

 Please indicates what the acronym LEI defines at the very beginning of the article.

 It is worthwhile to know, in a table, how many semi-structured interviews have been carried out, but in general, I have some doubts about this. It seems that six interviews have been conducted, and if so, I think that generalizations are hard to develop. Please substantiate in the methods how the outcomes of the interviews have been operationalized, otherwise the paper will results a bit fragile from a methodological side.

 In this respect, I suggest to extend the sub-section "data analysis" to address the need of further details of methods and analysis.

 Also, it would be good to know what was the benefit of using Sonix.ai.

 In general, I think that the paper does not achieve the aim - illustrated in the Abstract - of "gaining insights into the ways that a more balanced approach can be achieved, by taking into consideration the (mutual) interests, barriers and expectations of the municipality and local citizen initiatives."

 I think the aim should be recalibrate according to the empirical effort that has been carried out. 

 I suggest to better introduce the results and describe how the main topics raised from the qualitative analysis have been deployed. At the current form, these topics are merely summarized without a proper introduction to the discussion.

 I suppose that this paper - according to its form of "brief report" - is dedicated to policy makers to provide some policy recommendations, as it does not fulfill several criteria for being considered an academic article. The theoretical framework is too concise, the presentation of methods is too synthetic, and the discussion of the outcomes is a bit disentangled from the rest of the analysis. However, the paper can still be significant for providing policy recommendations, and I think that this aim should be clearly stated in the introduction. If the aim was different, I think that paper should be densely reframed and reorganized from the abstract. The very aim of gaining insights into a more balanced approach between top-down and bottom-up should be better presented according to the contents of the paper, and this is currently not the case.

 Based on this report, I think that the paper deserves some revisions before indexing. I suggest an approval with reservation, but I stress that report needs a revision of both the theoretical framework and the ways in which the outcomes of the qualitative analysis are presented, to better connect theory and empirical activities.

 Below, I suggest some citations that could be useful to the authors.

If applicable, is the statistical analysis and its interpretation appropriate?

Not applicable

Are all the source data and materials underlying the results available?

Yes

Is the study design appropriate and is the work technically sound?

Yes

Is the work clearly and accurately presented and does it engage with the current literature?

Partly

Are the conclusions drawn adequately supported by the results?

Partly

Are sufficient details of methods and analysis provided to allow replication by others?

No

Reviewer Expertise:

Qualitative research, local welfare, energy transition, LEIs, energy poverty, renewable energy communities.

I confirm that I have read this submission and believe that I have an appropriate level of expertise to confirm that it is of an acceptable scientific standard, however I have significant reservations, as outlined above.

## References

[1] : An advocacy coalition framework of policy change and the role of policy-oriented learning therein. *Policy Sciences* .1988;21(2-3) : 10.1007/BF00136406 129-168 10.1007/BF00136406

[2] : Testing the social, organizational, and governance factors for success in local low carbon energy initiatives. *Energy Research & Social Science* .2019;58: 10.1016/j.erss.2019.101269 10.1016/j.erss.2019.101269

[3] : Local civil society based renewable energy organisations in the Netherlands: Exploring the factors that stimulate their emergence and development. *Energy Policy* .2014;69: 10.1016/j.enpol.2014.01.046 297-307 10.1016/j.enpol.2014.01.046

[4] : Methods and tools for community energy planning: A review. *Renewable and Sustainable Energy Reviews* .2015;42: 10.1016/j.rser.2014.11.042 1335-1348 10.1016/j.rser.2014.11.042

[5] : Power to the people: local energy initiatives as seedbeds of innovation?. *Energy, Sustainability and Society* .2014;4(1) : 10.1186/2192-0567-4-2 10.1186/2192-0567-4-2

[6] : Towards a typology of intermediaries in sustainability transitions: A systematic review and a research agenda. *Research Policy* .2019;48(4) : 10.1016/j.respol.2018.10.006 1062-1075 10.1016/j.respol.2018.10.006

[7] : Reconfiguring actors and infrastructure in city renewable energy transitions: A regional perspective. *Energy Policy* .2021;158: 10.1016/j.enpol.2021.112544 10.1016/j.enpol.2021.112544

# Response

**Published:** 2024-10-10

**Authors:** Ifigenia Psarra

**Affiliations:** Noorderruimte Research Center, Hanze University of Applied Sciences, Groningen, The Netherlands

Thank you very much for your insightful feedback and recommendations. We appreciate the time and effort you put into reviewing our brief report and have addressed your comments as follows:

- We have now introduced the framework in the introduction to provide clearer context and structure.
- We would like to kindly clarify that the format of a brief report emphasizes the inclusion of only "essential references." As such, we have adhered to this guideline, but we understand the importance of the broader literature. Consequently, we have added a few additional relevant articles to the reference list.
- While we recognize the extensive literature on community energy, we believe that a more comprehensive discussion would exceed the scope of a brief report. Nevertheless, we have enriched our references where possible.
- As this is a qualitative study, the objective was not to maximize the number of interviews, but rather to ensure that key perspectives from representatives of the main energy initiatives were captured. Our case study focused on the municipality of Groningen, and apart from the LEIs representatives, we also interviewed a municipal representative actively involved in local energy policies.
- We compared our results with existing literature, especially referencing the TNO (2020) report, to identify if similar needs are experienced by LEIs in different contexts or cities. 
- Lastly, as noted above, a deep theoretical discussion would exceed the format of this brief report, which is designed to highlight empirical findings and their implications.

We have endeavored to comply with the specific guidelines of the brief report format, which emphasizes conciseness in presenting essential empirical data, as outlined in the Open Research Europe guidelines. https://open-research-europe.ec.europa.eu/for-authors/article-guidelines/social-sciences/brief-report/ Thank you again for your valuable feedback, and we hope our revisions address your concerns.

# Reviewer report: Reviewer response for version 1

**DOI:** 10.21956/openreseurope.18838.r42520

**Published:** 2024-08-10

**Authors:** Marie Claire Brisbois

**Affiliations:** 1 University of Sussex, Brighton, England, UK

The article reports on a study into barriers to community energy initiatives in Groningen, NL, and provides recommendations based on the findings. Findings are based on six interviews with local energy groups, the municipality, and a local intermediary. 

 The topic is interesting but it would have been very helpful to have more discussion of the theoretical approach. The abstract discusses an "implementation analysis" framework but this is not discussed in the text of the article. Text providing theoretical background and a discussion of the strengths and weaknesses of this approach would have been very helpful. Right now, the only links to academic literature appear in the Introduction and this thus reads more like a consulting report than an academic article. There is nothing wrong with consulting reports, and they are often very useful in providing insights for improving local energy activities. However, they are not academic articles which need clear theoretical background, robust evidence, and some form of Discussion section which links the findings to wider knowledge on the topic. The literature actually has a lot to say about supporting local energy groups and it would be very helpful to situate the findings of this study within that broader knowledge.

 Regarding the evidence, the data pool is very small and a lot of weight is put on a single interview from the municipality - as this represents the "top down" perspective. While a small sample can be appropriate in certain situations, this study appears to make claims about generalisability. For claims about generalisability, even if it's just to the North of the Netherlands (i.e. outside of the case study site), it would be good to have more robust data. Robustness could have been improved through more interviews at the "top" level, a document analysis, and also through links to wider literature and other contexts. I appreciate that there may not be many people working on local energy in this context, and it would therefore be difficult to secure other interviewees. With such a limited sample, it becomes very important to explore whether the results found here are consistent with those elsewhere, and whether lessons can be learned.

 It also would have been nice to have a bit of reflection on the framework used. Was this useful for examining this context? Was anything important missed? Would you add anything?

 In general, this looks like an interesting set of findings that will be very useful for the municipality but, to be published academically, the article needs to be developed as an academic article with sections establishing theoretical background and approach, and some kind of discussion section. Data robustness needs to be improved as well.

If applicable, is the statistical analysis and its interpretation appropriate?

Not applicable

Are all the source data and materials underlying the results available?

Yes

Is the study design appropriate and is the work technically sound?

Partly

Is the work clearly and accurately presented and does it engage with the current literature?

No

Are the conclusions drawn adequately supported by the results?

Yes

Are sufficient details of methods and analysis provided to allow replication by others?

No

Reviewer Expertise:

energy transitions, community energy, energy governance

I confirm that I have read this submission and believe that I have an appropriate level of expertise to state that I do not consider it to be of an acceptable scientific standard, for reasons outlined above.

# Response

**Published:** 2024-10-10

**Authors:** Ifigenia Psarra

**Affiliations:** Noorderruimte Research Center, Hanze University of Applied Sciences, Groningen, The Netherlands

Thank you very much for your valuable feedback and thoughtful suggestions. We appreciate your insights, and have made the following revisions to the brief report:

- We see your point regarding the theoretical section. However, as the format of the brief report does not include an in-depth theoretical discussion, we have adhered to its structure as per the guidelines: Brief Report Guidelines.
- We have incorporated additional scientific articles that discuss top-down and bottom-up approaches, and we have added references related to the role of intermediaries to enrich the discussion.
- Thank you for pointing out the need to define the acronym LEI. We have now clarified this at the beginning of the article.
- We have expanded the explanation of our method and included a table listing the LEIs that participated in the interviews. 
- The results of our analysis have been grouped and explained in greater detail. We included a brief yet accurate description of our analysis method, in line with the brief report format, and referenced further readings for more extensive information.
- Sonix was the tool we used to transcribe interviews (that is now clearly explained in the methods section).
- We have reformulated the research aims to align more closely with the study’s outcomes. We reviewed our recommendations carefully to ensure they highlight specific actions that balance the top-down and bottom-up approaches.
- An introductory paragraph has been added to the results section to provide better flow.
- We appreciate your suggestion regarding policy recommendations and have placed more emphasis on them in our revisions.
- While we followed your recommendation to add additional references, we were mindful of the word limit imposed by the brief report format. Therefore, we ensured that the theoretical and methodological sections remain concise, as per the format's requirements.
- Finally, we have updated and clarified our research aim in both the abstract and the introduction. We also further categorized the results section for clarity. We hope these changes have enhanced the quality of the report and made it suitable for indexing in ORE.

Thank you again for your invaluable feedback.
